# A Novel Algorithm to Analyze Multi-Frequency Electrocochleography Measurements to Monitor Electrode Placement During Cochlear Implant Surgery

**DOI:** 10.3390/brainsci14111096

**Published:** 2024-10-30

**Authors:** Eric E. Babajanian, Kanthaiah Koka, Aniket A. Saoji

**Affiliations:** 1 Department of Otolaryngology-Head and Neck Surgery, Mayo Clinic, Rochester, MN 55902, USA; babajanian.eric@mayo.edu; 2 Advanced Bionics, Valencia, CA 91355, USA; kanthaiah.koka@advancedbionics.com

**Keywords:** cochlear implants, cochlear microphonics, electrocochleography

## Abstract

Objectives: During cochlear implant (CI) electrode placement, single low-frequency (e.g., 500 Hz) cochlear microphonics (CM) measurements are used to monitor hair-cell function and provide feedback to avert insertion trauma. However, it can be difficult to differentiate between trauma and the electrode’s progression through the cochlea when monitored with a single frequency. Multi-frequency CM measurements, while more complex to analyze, can provide more accurate feedback by measuring CM from various locations along the basilar membrane. Methods: A new algorithm was developed to analyze multi-frequency CM tracings by comparing amplitude and phase changes across different test frequencies. The new algorithm was evaluated as to its ability to identify drop-alarm instances with the multi-frequency approach, as compared to single-frequency 500 Hz tracings. Results: The algorithm presented in this manuscript uses the relationship between CM amplitude and phase changes across frequencies to provide real-time feedback during CI electrode placement. The results show that multi-frequency CM tracings raised an alarm only 0.5 times, as compared to 2.8 instances of alarm raised for the single-frequency 500 Hz CM measurements. Conclusions: Multi-frequency CM tracings can help reduce the number of alarms which may be false positives prompting unnecessary electrode manipulations, thereby minimizing the risk of insertion trauma.

## 1. Introduction

Cochlear implantation is the current gold-standard treatment for patients with advanced sensorineural hearing loss. With expanding criteria for implantation, it is not uncommon for patients to present with a high-frequency hearing loss and measurable residual hearing in the lower frequencies. Residual hearing preservation has important postoperative implications for the patient, including the potential for electroacoustic stimulation [1,2]. When consenting for cochlear implant (CI) surgery, it is important to discuss the fact that preoperative residual hearing may be lost in the form of decreased pure-tone thresholds because of the surgery. Ideally, the entire cochlear implant electrode array should be inserted into the scala tympani to achieve the best possible postoperative sound and speech perception outcomes [3,4]. Trauma to the inner ear because of CI electrode placement, however, is thought to be an important variable contributing to the loss in preoperative residual hearing [4,5,6].

Electrocochleography (ECochG) allows the measurement of a physiologic response from the inner ear to an acoustic stimulus, and has been increasingly utilized intraoperatively at the time of cochlear implantation with the goal of hearing preservation [7]. ECochG offers the ability to obtain real-time intraoperative feedback regarding CI electrode placement and potential intracochlear trauma [8,9,10,11]. The cochlear microphonic (CM) is a component of the ECochG waveform that reflects movement of the outer ear hair cells and can be recorded during CI electrode placement. Typically, a single low-frequency pure-tone stimulus such as 250 or 500 Hz is used to elicit CM, with a characteristic place closer to the apical end of the cochlea. The most apical electrode on the implant array is used as the recording electrode. Given that the most apical electrode approaches the characteristic place of stimulation during insertion, the CM amplitude tends to increase with implant insertion [12,13]. The amplitude of the CM may decrease, either due to interference, inner ear trauma, or advancement past the characteristic place, or to passing basal activation of tuning curve, raising an “alarm” when utilizing ECochG during electrode insertion [10,14,15]. The primary objective of CM monitoring is to rapidly detect any decrease in CM amplitude and immediately notify the surgeon. This timely warning allows for course corrections in electrode placement, minimizing the risk of inner ear trauma. CM amplitude monitoring can be performed by (1) a trained clinician who manually observes changes in CM amplitude and alerts the surgeon or (2) an algorithm that provides continuous auditory feedback and raises an alarm when a drop in CM amplitude is detected. A decrease in CM amplitude might be due to either electrode insertion trauma or because of the advancement of the recording electrode through the cochlear space. False positives can lead to unnecessary surgical manipulations, potentially increasing the risk of trauma.

Multi-frequency ECochG (electrocochleography) offers a promising solution to the challenge of false alarms in CM monitoring [16,17]. With multi-frequency ECochG, the stimulus is delivered via a pure-tone complex consisting of three or four frequencies to elicit CMs along the tonotopic axis of the cochlea. When CM amplitude decreases across all test frequencies, it is a strong indicator of cochlear trauma. However, if the amplitude decreases at one frequency but increases at others, it is more likely due to the electrode’s movement through the cochlear space. While multi-frequency ECochG offers significant advantages in CM monitoring, it presents a challenge in visual analysis. Even for experienced clinicians, interpreting four or five CM tracings simultaneously and providing immediate feedback to the surgeon can be demanding. To address the limitations of visual multi-frequency ECochG analysis, a new algorithm was developed. This algorithm analyzes both amplitude and phase changes in the CM measurements from multiple frequencies. By providing continuous auditory feedback to the surgeon and raising an alarm when the data indicate potential electrode insertion trauma, the algorithm helps minimize false positives and reduce the risk of unnecessary surgical manipulations. The multi-frequency CM measurement reported by Saoji et al. in 10 CI patients was used to develop this algorithm [16]. This study elaborates on the decision algorithm and compares multifrequency CM measurements to single-frequency (500 Hz) CM tracings. The results of this analysis can improve feedback and improve outcomes by decreasing false alarms and unnecessary electrode manipulations.

## 2. Methods

Ten patients (6 male and 4 female) with advanced sensorineural hearing loss and an average age of 73.1 years (SD = 10.4) were included in the present study after preoperative evaluation confirmed their CI candidacy. All patients underwent cochlear implantation using a HiRes Ultra 3D CI from Advanced Bionics (Valencia, CA, USA). All patients underwent cochlear implantation using a standard approach via round window insertion using an Advanced Bionics HiFocus SlimJ electrode array. All subjects eventually received a full electrode insertion. 

The OM Suite software on an AIM tablet (AIM^TM^ System, Version 1.2R, Advanced Bionics LLC, Valencia, CA, USA) was modified (AIM1.2R) to allow simultaneous presentation of multi-frequency tone burts (up to 4 tones) and record CMs. The most apical electrode was used as the recording electrode. ECochG measurements were successfully measured throughout electrode placement. CM amplitude and phases were extracted using fast Fourier spectrum analysis for each stimulus frequency.

A new ECochG amplitude drop-alarm decision algorithm was developed based on multi-tone ECochG measurements. The decision algorithm considered both CM amplitude and phase across the multifrequency CM tracings. To assess whether a decrease in CM amplitude at one test frequency is indicative of a broader pattern, the amplitude tracings from other frequencies are compared. If a similar drop is observed across multiple frequencies, it suggests a more generalized decline. Additionally, phase change is correlated across frequencies when a CM amplitude decrease is noted in one or more test frequencies. The AIM software (version 1.0) allows CM amplitude drop as a parameter but not phase change. An amplitude drop of 6 dB and phase change of 180 degree have been used for the current algorithm, but amplitude drop is selectable parameter.

**Figure 1:**
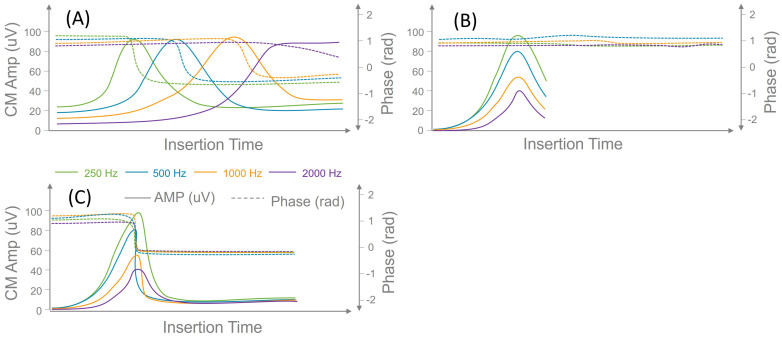
Schematic illustration of CM amplitude and phase changes that can be observed during cochlear implant electrode placement. Panel (**A**) shows CM amplitude and phase changes at different time points during electrode placement. Panel (**B**) demonstrates a correlated decrease in CM amplitude across the different test frequencies but with minor phase shifts of less than 180 degrees. Panel (**C**) illustrates a correlated decrease in multifrequency CM amplitude accompanied by a large phase change (≥180 degrees).

Figure 1 schematically illustrates CM amplitude and phase changes that may be associated with electrode insertion trauma (Panel C) or due to the advancement of the recording electrode beyond the characteristic place of CM along the cochlear space (panel A). Each panel plots insertion time on the *x*-axis, CM amplitude (solid lines) on the left *y*-axis, and corresponding phase (dotted lines) changes on the right *y*-axis. 

Panel A depicts multifrequency CM amplitude and phase values consistent with cochlear tonotopicity and the absence of electrode insertion trauma. Here, the CM tracing for a high-frequency stimulus (e.g., 2000 Hz) initially increases with electrode advancement before declining. Phase is also changed at the peak of the CM amplitude, measured at 2000 Hz. Conversely, CM amplitudes for the 250, 500, and 1000 Hz stimuli progressively increase. Each of these lower-frequency CM tracings eventually peaks and decreases at distinct insertion time points (except 250 Hz). Notably, a 180° (π radians) phase reversal accompanies the CM peak at each stimulus frequency, indicating electrode passage beyond the CM generation site [12,18]. This pattern shows an amplitude decrease and corresponding phase changes at different time points during the advancement of the recording electrode beyond the characteristic place for CM generation along the basilar membrane. Therefore, no alarm is raised when such a pattern is measured during CI electrode placement.

Panel B demonstrates a correlated decrease in CM amplitude across the different test frequencies but with minor shifts of less than 180 degrees. This multifrequency CM tracing pattern, characterized by a correlated amplitude decline without accompanying phase changes (or with changes of less than 180 degrees), suggests the electrode may be passing through a region of hair cells or the basal tails of tuning curves. This scenario is considered non-traumatic, and no alarm is triggered. It is noteworthy that Koka et al., focusing on single-frequency and phase analysis, classified a drop in amplitude without phase change as a traumatic event [13]. However, our multi-frequency tracings allow for a more nuanced interpretation, treating this pattern as non-traumatic due to the additional information provided by multiple frequencies.

Panel C illustrates a correlated decrease in multifrequency CM amplitude accompanied by a large phase change that is more than 180 degrees. This pattern, characterized by a drop across multiple frequencies and a significant phase shift, is indicative of electrode trauma or passage through the basilar membrane. This scenario is distinct from Panel A, in which each frequency exhibits phase reversal at different locations. In contrast, the consistent, large phase shift across all frequencies in Panel C suggests a potential impact on the basilar membrane. This is considered a traumatic change and triggers an alarm.

The multi-frequency ECochG alarm decision algorithm outlined previously was applied to the data presented by Saoji et al. [16]. This analysis aimed to distinguish between single-frequency alarms potentially indicative of electrode insertion trauma and those signaling the electrode’s progression beyond the cochlear region where CM is generated.

## 3. Results

**Figure 2:**
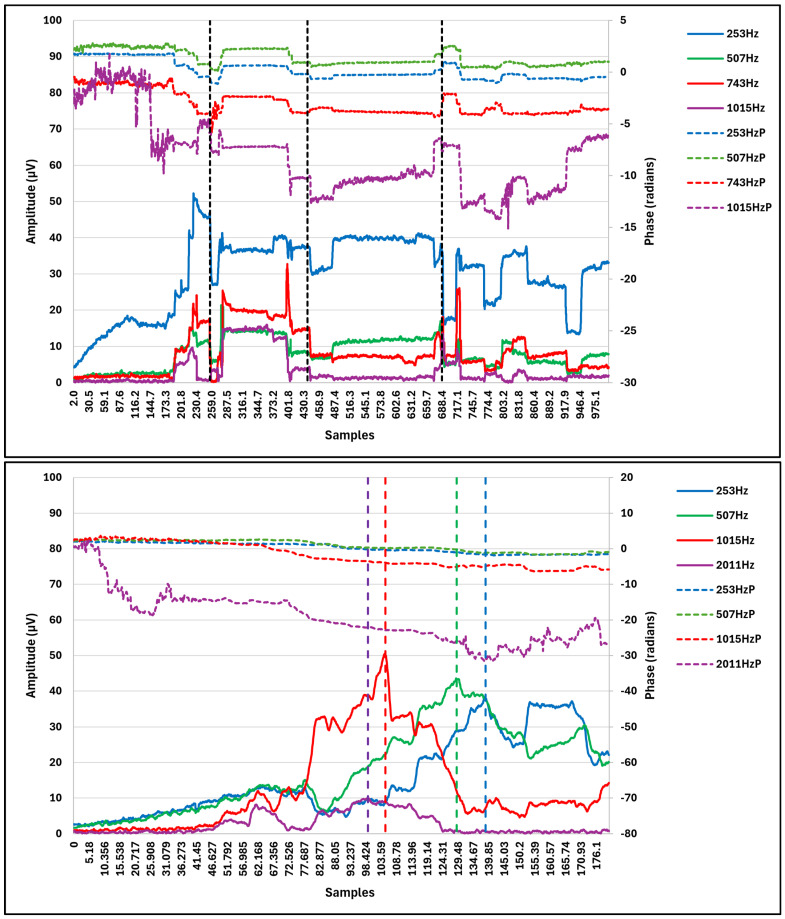
Two illustrative cases of multi-frequency CM tracings from the cohort of 10 patients reported by Saoji et al. These tracings depict the amplitudes and phases of CM responses across four test frequencies. In the **top** panel, the tracings show three instances of a correlated decrease in CM amplitude across all frequencies, accompanied by phase changes of over 180 degrees (dashed lines). This pattern is indicative of electrode insertion trauma. The **bottom** panel shows decreases in CM amplitude at various time points (dashed lines); the phase changes do not consistently align with the amplitude reductions. This pattern suggests that the changes are more likely due to the electrode’s movement through the cochlear space and do not indicate trauma.

Figure 2 presents CM amplitude and phase tracings for two representative patients (CI5 and CI6) from the 10-patient cohort described by Saoji et al. [16]. The upper panel displays data associated with CI5, while the lower panel presents data associated with CI6. CM amplitude traces are depicted with solid lines, and the corresponding phase curves are represented by dotted lines, maintaining the same color scheme for consistency.

The CI5 data demonstrate CM amplitude and phase variations across four frequencies during insertion. Three significant events are marked, each characterized by a decrease in CM amplitude across multiple frequencies and substantial phase changes (exceeding 3.14 radians, or 180 degrees) across all frequencies. These events closely resemble the pattern observed in Figure 1, Panel C, suggesting traumatic occurrences. The decision algorithm classified these events as traumatic, resulting in presentation of a drop alarm.

The bottom panel presents CM amplitude and phase variations across four frequencies during electrode placement for CI6. Four significant events are marked. At the first event marker, CM amplitude and phase changes are observed only for 2011 Hz, indicating the electrode passing through the characteristic frequency location for this frequency. This single-frequency change is considered non-traumatic and does not trigger an alarm. Similar patterns are observed at the second, third, and fourth event markers, with CM amplitude and phase changes occurring at 1015 Hz, 507 Hz, and 253 Hz, respectively. These events are also interpreted as single-frequency changes and are classified as non-traumatic without an alarm.

For the cohort of 10 patients described by Saoji et al., the multifrequency algorithm outlined in the methods section was employed to determine the number of instances where a decrease in CM amplitude and phase change would be classified as electrode insertion trauma, triggering a drop alarm that prompts for electrode manipulation. These multifrequency-identified drop alarms were compared with single-frequency (at 500 Hz, as used in other studies and as the default setting in AIM software) amplitude-only alarms (see Table 1). On average, 2.8 instances were detected for the single-frequency CM tracings and 0.5 instances were detected for the multi-frequency tracings. These results show that single-frequency 500 Hz CM data generated significantly more alarms than were generated with multifrequency alarms (including potentially false alarms), increasing the risk of inadvertent electrode manipulation, and ultimately compromising cochlear health and CI performance. Monitoring CM signals from multiple locations along the basilar membrane in the cochlea provided more comprehensive information, aiding in the differentiation between atraumatic and traumatic events. This approach enables more accurate assessments of electrode insertion trauma and minimizes the number of false alarms, given the number that would otherwise occur if single-frequency CM tracing were used for intraoperative monitoring.

## 4. Discussion

During CI electrode placement, a low-frequency pure-tone stimulus (e.g., 500 Hz) is used to monitor hair-cell function and detect CM. A decrease in CM amplitude is considered a potential sign of cochlear trauma and can guide the surgeon in adjusting electrode placement. However, using a single-frequency measurement makes it difficult to differentiate between trauma and the electrode’s progression through the cochlea in real time. Multi-frequency ECochG measurements, while more complex to analyze visually, can provide more precise information by measuring CM from various locations along the basilar membrane. 

The algorithm presented here analyzes multi-frequency ECochG data collected during CI electrode placement. By comparing amplitude and phase changes across different test frequencies, it differentiates between electrode insertion trauma and the recording electrode’s progression through the cochlea. A significant decrease (more than 6 dB) in CM amplitude across frequencies (more than 2 frequencies) is flagged as potential trauma if it is accompanied by a correlated phase shift (more than 180 degrees). However, if the amplitude decrease occurs without a corresponding phase change (less than 180 degrees), it is likely indicative of the electrode advancing through a patch of hair cells. - A decrease in CM amplitude occurring in fewer than two frequencies during a high-to-low frequency analysis, and linked to a phase shift exceeding 180 degrees, is regarded as an indication of an electrode passing the characteristic frequency location and is classified as non-trauma.

The algorithm was tested on multi-frequency ECochG recordings from Saoji et al. [16] and compared to single-frequency 500 Hz CM tracings. The results showed that the new multi-tone ECochG algorithm can decrease drop alarms significantly (mean 2.8 vs. mean 0.5, n = 10) compared to single-frequency-based drop alarms. This algorithm could provide more accurate feedback in real time, reducing the risk of unnecessary electrode manipulation and potential trauma, based on the number of false trauma alarms encountered in the single-frequency context. While the novel algorithm shows promise in decreasing the number of alarms (potentially false trauma alarms) from electrode movement, further validation is necessary. Testing the algorithm on a larger dataset with postoperative outcomes and correlating the results with postoperative CT scans will provide valuable insights into its clinical utility. By comparing the algorithm’s predictions with the actual electrode placement, clinicians can assess the algorithm’s accuracy in identifying cochlear trauma.

## 5. Conclusions

Multi-frequency CM monitoring can help prevent unnecessary electrode manipulation and reduce the risk of trauma. However, they may not be effective for patients with limited residual hearing. The algorithm described here can analyze multi-frequency CM data in real time, providing valuable feedback to surgeons for optimized CI electrode placement.

## Figures and Tables

**Table 1:** Quantification of occurrences in which declines in 500 Hz and multi-frequency cochlear microphonic (CM) measurements are attributed to electrode insertion trauma during cochlear implant surgery.

Subjects	500 Hz	Multi-Frequency
CI1	4	0
CI2	7	0
CI3	0	0
CI4	2	0
CI5	5	1
CI6	0	0
CI7	2	2
CI8	2	2
CI9	3	0
CI10	3	0

## Data Availability

No new data were created or analyzed in this study. The present study describes an algorithm designed to analyze multi-frequency ECochG tracings. The data used to validate the algorithm was reported by Saoji et al. [16].
